# Understanding Successful Aging: The Power of Growth Mindsets and Openness to Experience

**DOI:** 10.1093/geroni/igaf122.2481

**Published:** 2025-12-31

**Authors:** Jessica Fleck, Katherine Wilkinson, Tanya Sharma, Mackenzie Unsworth, Ava Marie Cacia, Claire Jacobus, Janina Costello

**Affiliations:** Stockton University, Galloway, New Jersey, United States; Stockton University, Galloway, New Jersey, United States; Stockton University, Galloway, New Jersey, United States; Stockton University, Galloway, New Jersey, United States; Stockton University, Galloway, New Jersey, United States; Stockton University, Galloway, New Jersey, United States; Stockton University, Galloway, New Jersey, United States

## Abstract

Successful aging has evolved beyond merely maintaining physical health and independence to encompass life satisfaction and overall well-being. Consequently, the elements that may contribute to successful aging have expanded to include personality traits, such as a growth mindset and openness to experiences. A growth mindset refers to the belief that an individual’s actions and efforts are more influential than predetermined factors, such as age or genetics, in determining outcomes. Openness, on the other hand, reflects a person’s willingness to consider new ideas and experiences and may promote successful aging, as individuals high in openness are more likely to engage in a diverse range of activities, which helps build cognitive reserve. We examined the roles of growth mindset and openness in successful aging among adults aged 60 to 80 years. We hypothesized that greater openness would be linked to increased engagement, and that both openness and growth mindset would predict cognitive and emotional aspects of successful aging. Our findings supported this hypothesis, showing that higher levels of openness and a growth mindset were associated with better aging outcomes, including improved emotion regulation and attention regulation. However, when we included both openness and growth mindset as predictors in a regression model of successful aging, only growth mindset emerged as a significant predictor. Mediation analyses revealed that openness mediated the relationship between years of education and growth mindset, and that growth mindset mediated the relationship between openness and successful aging. Our research supports the role of personality and mindsets in aging outcomes.

